# *Porphyromonas gingivalis* and Its Systemic Impact: Current Status

**DOI:** 10.3390/pathogens9110944

**Published:** 2020-11-13

**Authors:** Feng Mei, Mengru Xie, Xiaofei Huang, Yanlin Long, Xiaofeng Lu, Xiaoli Wang, Lili Chen

**Affiliations:** 1 Department of Stomatology, Union Hospital, Tongji Medical College, Huazhong University of Science and Technology, Wuhan 430022, China; hust_mei@hust.edu.cn (F.M.); hbxiemengru@hust.edu.cn (M.X.); fffly@hust.edu.cn (X.H.); hugh_long1993@hust.edu.cn (Y.L.); lxf_yuki@hust.edu.cn (X.L.); 2 Hubei Province Key Laboratory of Oral and Maxillofacial Development and Regeneration, Wuhan 430022, China; 3 Department of Obstetrics and Gynecology, Union Hospital, Tongji Medical College, Huazhong University of Science and Technology, Wuhan 430022, China

**Keywords:** *Porphyromonas gingivalis*, periodontitis, systemic diseases

## Abstract

The relationship between periodontitis and systemic diseases, notably including atherosclerosis and diabetes, has been studied for several years. *Porphyromonas gingivalis*, a prominent component of oral microorganism communities, is the main pathogen that causes periodontitis. As a result of the extensive analysis of this organism, the evidence of its connection to systemic diseases has become more apparent over the last decade. A significant amount of research has explored the role of *Porphyromonas gingivalis* in atherosclerosis, Alzheimer’s disease, rheumatoid arthritis, diabetes, and adverse pregnancy outcomes, while relatively few studies have examined its contribution to respiratory diseases, nonalcoholic fatty liver disease, and depression. Here, we provide an overview of the current state of knowledge about *Porphyromonas gingivalis* and its systemic impact in an aim to inform readers of the existing epidemiological evidence and the most recent preclinical studies. Additionally, the possible mechanisms by which *Porphyromonas gingivalis* is involved in the onset or exacerbation of diseases, together with its effects on systemic health, are covered. Although a few results remain controversial, it is now evident that *Porphyromonas gingivalis* should be regarded as a modifiable factor for several diseases.

## 1. Introduction

Chronic periodontitis, a multifactorial chronic inflammatory disease resulting from dysbacteriosis, is characterized by the destruction of connective tissue and alveolar bone, and it has become the primary reason for tooth loss in adults. It affects nearly 50% of the population worldwide, representing one of the most common inflammatory diseases in humans [1]. Over the past two decades, mounting evidence has supported periodontitis as a potential risk factor for multiple systemic diseases, for example, cardiovascular diseases. As the key etiological agent in periodontitis, *Porphyromonas gingivalis* (*P. gingivalis*) has proved to be closely correlated with the occurrence and development of many systemic diseases, such as atherosclerosis, cancer, and Alzheimer’s disease [2,3,4]. The roles of *P. gingivalis* in systemic diseases have been discussed for several years, and several reviews have summarized the effects and the inner mechanisms [5,6,7,8,9,10]. However, most of these articles only focus on the relationship between *P. gingivalis* and one specific disease or one class of diseases. A global understanding of the effect of *P. gingivalis* on overall health is lacking. In this review, we systematically and comprehensively provide an update and summary of the literature on *P. gingivalis*-related systemic diseases that affect the whole body, as well as the internal mechanisms, to provide a more comprehensive understanding of *P. gingivalis* and its relationship with systemic diseases.

### Characteristics of P. gingivalis

*P. gingivalis*, one of over 700 bacterial species in the oral cavity, is a Gram-negative, anaerobic, rod-shaped bacteria that forms black colonies on blood agar and requires the presence of heme or hemin and vitamin K in its growth milieu. It is a successful colonizer of the oral epithelium and an important component of subgingival microbiomes [11]. *P. gingivalis* is responsible for the chronic form of periodontitis, as it can remodel the commensal bacterial community to promote a state of dysbiosis [12]. Throughout evolution, it has developed unique and intricate mechanisms, such as the alteration of signaling pathways of inflammation, the complement system, the cell cycle, and apoptosis, and the interaction with various host receptors, thereby engineering its environment or modifying the host’s immune response to modulate the entire ecosystem and to persist in host tissues [13]. The survival strategies and pathogenicity of *P. gingivalis* largely depend on its diverse virulence factors, including its own structural components (lipopolysaccharide, fimbriae, heat shock proteins, etc.) and secretory components (gingipains and outer membrane vesicles).

Fimbriae are crucial for enabling *P. gingivalis* to specifically bind to eukaryotic cells and other species of bacteria to enhance bacterial motility, biofilm formation, and bacterial invasion of the cells [14]. It can also activate various host cells and subvert host immune clearance [14]. *P. gingivalis* lipopolysaccharide (LPS) can trigger the innate immune response via activation of Toll-like receptors (TLRs) [15]. The lipid A component of *P. gingivalis* LPS exhibits two predominant variations of acylation that are attributed to different strains and microenvironmental conditions: the penta-acylated LPS activates TLR4, while tetra-acylated LPS acts as a TLR4 antagonist and TLR2 agonist [15]. The heat shock protein 60 (HSP60) component of *P. gingivalis* is remarkably immunogenic and plays a critical role in *P. gingivalis*-induced autoimmune diseases [16]. Gingipains, which consist of lysine-gingipain (Kgp) and arginine-gingipain (Rgp), have multiple impacts on both innate and acquired immunity. These enzymes play essential roles in host colonization, host defense deactivation, tissue destruction, and nutrient acquisition [17]. Outer membrane vesicles (OMVs) from *P. gingivalis* are enriched in major virulence mediators, such as gingipains, LPS, and the capsule, and participate in biofilm development, host interaction, colonization, and immune defense evasion [18]. Moreover, its characteristic features, such as concentrated gingipains, together with its ability to travel to distant sites, might participate in *P. gingivalis*-associated systemic disorders [19].

*P. gingivalis* in local periodontal tissue can enter the vasculature through ulcerated epithelium [20] and lymph vessels [21] shortly after everyday activities, such as brushing and chewing, along with dental procedures. Some studies have indicated that *P. gingivalis* can survive in other organs besides the oral cavity. Viable *P. gingivalis* has been detected in human atherosclerotic plaque tissues [22] and mouse lungs [23] through tissue homogenates that were incubated with cells or cultured directly on blood agar plates. Cellular experiments have also provided evidence for the survival of *P. gingivalis* in some cells. For example, live *P. gingivalis* has been isolated from human aortic endothelial cells [24], human pancreatic tumor cells [25], and human myeloid dendritic cells [26]. All of the properties described above confer this species with the ability to invade distant tissues, where it is then involved in the onset and/or progression of systemic diseases.

## 2. Cardiology

Cardiovascular diseases (CVDs) are a cluster of conditions that involve the heart and blood vessels, and they remain the most common cause of death throughout the world. Considerable epidemiologic evidence has suggested that periodontal disease is a risk factor for CVDs [27]. For example, periodontal pathogens have been found in CVD plaques, and the detection rate of *P. gingivalis* has been as high as 100% [28]. The relationship between *P. gingivalis* and CVDs has been deeply studied in recent decades.

### 2.1. Atherosclerotic Cardiovascular Diseases (ACVDs)

ACVDs, including coronary artery disease and stroke, are a form of CVDs with high morbidity and mortality rates. Atherosclerosis (AS), resulting from the progressive accumulation of lipids, calcium, macrophages, and other components in the artery wall, is the pathological basis of ACVDs. Much evidence has linked periodontitis to an increased risk of AS [27]. Periodontal treatment has been shown to improve the endothelial function and reduce the biomarkers (such as CRP, IL-6, TNF-α, fibrinogen, triglycerides) of atherosclerotic disease, particularly in individuals already suffering from CVDs [29]. Trials in humans have found *P. gingivalis* in clinical samples at a detection rate of 82.61% by fluorescent in situ hybridization assay [30]. Subsequently, in vivo experiments confirmed the promoting effect of *P. gingivalis* on AS. Infection with *P. gingivalis* exacerbated atherogenesis in apolipoprotein E (ApoE)-deficient mice [2,30], and the proximal aortic lesion size in *P. gingivalis*-inoculated mice was 2-fold larger than that in control mice [2]. These results strongly suggest that *P. gingivalis* may enter the lesion area and promote the development of AS.

**Figure 1:**
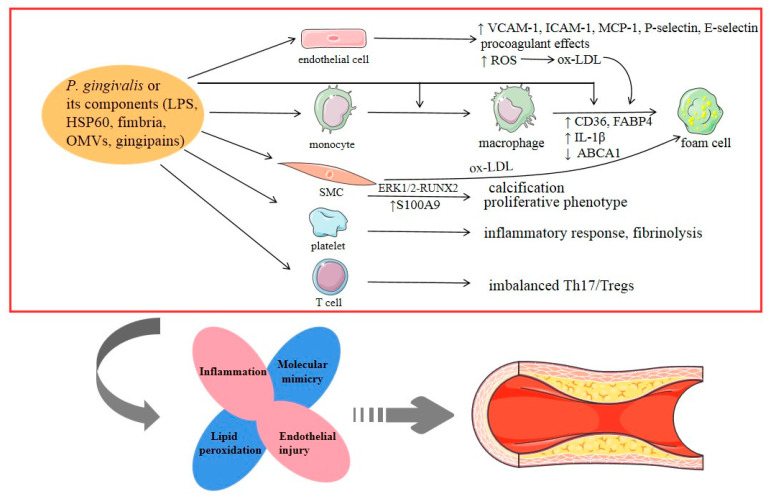
*Porphyromonas gingivalis*-induced stimulation of accelerated atherosclerosis. The possible underlying mechanisms can be summarized as follows: (i) inflammation, (ii) endothelial injury, (iii) lipid peroxidation, and (iv) molecular mimicry. ox-LDL: oxidized low-density lipoprotein.

Most scholars regard AS as an excessive inflammatory response after arterial wall endothelial dysfunction resulting from many damage factors. The pathogenesis mechanisms of AS are highly complex and include the activation of endothelial cells and platelets, recruitment of leukocytes (mainly monocytes and macrophages), migration and proliferation of smooth muscle cells (SMCs), and formation of a lipid core, along with thrombosis and plaque instability. Studies have shown that *P. gingivalis* can promote AS by affecting the function of all of these cells (Figure 1). First, *P. gingivalis* can activate endothelial cells and induce endothelial dysfunction. As reported in a previous review, *P. gingivalis* invades endothelial cells via the autophagic pathway while suppressing apoptosis [31]. Through the NF-κB or p38 MAPK pathway, its fimbria and LPS positively upregulate the expression of various adhesion molecules in endothelial cells, such as vascular cell adhesion molecule-1, intercellular adhesion molecule-1, monocyte chemoattractant protein, P-selectin, and E-selectin [32,33]. This is an essential step in the pathogenesis of endothelial dysfunction. In recently published research, it was revealed that *P. gingivalis* enhanced oxidative stress and the inflammatory response in aortic endothelial cells via the NF-κB-BMAL1-NF-κB signaling loop, leading to the aggravation of AS [30]. Furthermore, *P. gingivalis* can induce procoagulant effects in endothelial cells, and this prothrombotic response may be associated with plaque progression and instability [34]. Second, it has been reported that IgG-opsonized *P. gingivalis* may bind to the FcγRIIa receptor on platelets and activate GPIIb⁄IIIa integrin, which becomes connected to Hgp44 adhesin through a fibrinogen bridge, inducing further platelet activation and aggregation [35]. A recent review analyzed the interactions of *P. gingivalis* with activated platelets, and the overall outcome may be the modified expression of cytokines (CKs), which might affect the inflammatory response and fibrinolysis [36]. Third, *P. gingivalis* and its virulence components (such as LPS, fimbria) are involved in each phase of monocyte activity during the formation of AS by supporting monocyte migration to the endothelial surface, intimal infiltration, and differentiation into pro-inflammatory macrophages and eventually foam cells [37,38]. Fourth, *P. gingivalis* can play a distinct role in foam cell formation, which is a critical step in the atherosclerotic process. *P. gingivalis* LPS promotes the accumulation of lipids in macrophages and the formation of macrophage-derived foam cells by upregulating CD36 (a scavenger receptor for low-density lipoprotein and oxidized low-density lipoprotein) as a result of c-Jun/AP-1 pathway activation and by downregulating ATP-binding cassette transporter A1 (cholesterol efflux moderator) due to increased calpain activity [38]. A study in 2019 experimentally determined that *P. gingivalis* promoted lipid uptake in macrophages by inducing the expression of fatty acid-binding protein 4, which may be dependent on the JNK pathway [39]. In addition, *P. gingivalis* enhanced the TLR2-CD36/SR-B2-dependent systemic release of IL-1β, leading to a subsequent increase in lipid uptake by macrophages and foam cell formation as a result of encountering IL-1β in the vessel wall [40]. Furthermore, the proteolytic activity of Rgp and Kgp was shown to induce lipid peroxidation and to change the expression of low-density lipoprotein and high-density lipoprotein, causing foam cell formation from macrophages [41]. Evidence from these studies suggests that lipoproteins play a key role in the connection between *P. gingivalis* and AS progression. *P. gingivalis* infection may also contribute to AS by modulating lipid metabolism and homeostasis. Fifth, vascular calcification is a prominent feature of AS. *P. gingivalis* LPS can stimulate the proliferation and calcification of SMCs, resulting in vascular calcification [42]. *P. gingivalis* OMVs were also reported to promote vascular SMC calcification through ERK1/2-RUNX2 [43]. Additionally, WADA et al. proposed a putative molecular mechanism in which *P. gingivalis* could contact or invade the SMC layer in blood vessels after endothelial cell injury and induce the expression of S100A9 (a member of the S100 calcium-binding protein family), which triggers the transformation of SMCs from a contractile to a proliferative phenotype, stimulating further cell growth and contributing to aortic intimal hyperplasia [9].

In addition to the mechanisms described above, molecular mimicry is also a hub that links *P. gingivalis* to AS. Endothelial cells express HSPs under some conditions, such as exposure to bacteria or CKs. *P. gingivalis* carries homologs to human HSPs, and *P. gingivalis* HSP60 antibodies cross-react with human HSPs [44]. In addition, T lymphocytes that react with both HSP60 from *P. gingivalis* and human HSPs have been found in the peripheral blood of CVD patients and in AS plaques [45]. Therefore, the immune response to *P. gingivalis* may cause cross-reactivity, resulting in endothelial damage and contributing to the pathogenesis of AS.

In the last two decades, accumulating evidence has supported the role of T cells as critical drivers and modifiers during AS progression. *P. gingivalis* infection, especially clones with type II fimbriae, has been found to decrease the population of regulatory T cells (Tregs) in AS patients [46]. Additionally, in a murine model with a larger lesion area and reduced plaque stability, *P. gingivalis* could influence T-cell differentiation and promote a pro-inflammatory Th17 response, suggesting that Th17/Treg imbalance plays a role during the process of AS [47]. This was also discussed in a previous review [5]. Moreover, the ability of *P. gingivalis* to manipulate components of the complement system, especially C5a, and antimicrobial peptides in remote locations may be implicated in AS development [10].

### 2.2. Myocardial Infarction (MI)

MI is the irreversible necrosis of cardiac cells and scar formation resulting from insufficient blood supply to the heart. Many studies have reported the promoting role of periodontitis in the development of MI. A large multicenter study showed that the risk of a first MI was markedly increased among periodontitis patients: The odds ratio was 1.28 after multi-variable adjustment [48]. Furthermore, analysis of thrombi collected from patients with acute MI showed the existence of periodontal pathogens. A recent meta-analysis that focused on the detection of periodontal bacteria in atherosclerotic plaque specimens from MI patients revealed that *P. gingivalis* was the most frequently detected species, with an average prevalence of 40% [49]. Further in vivo evidence proved that experimental *P. gingivalis* bacteremia induced myocarditis and/or MI in mice [50].

Researchers have attempted to explore the underlying biological mechanisms that link *P. gingivalis* and MI. Firstly, *P. gingivalis* participates in the progression of MI through direct action with cardiomyocytes. It promotes cardiomyocyte apoptosis by activating Bax and increases matrix metalloproteinase-9 (MMP-9) activity by enhancing oxidative stress, thereby exerting a strongly detrimental effect on the healing process of the infarcted myocardium and subsequently leading to cardiac rupture after MI [51]. Additionally, *P. gingivalis* may aggravate myocardial injury after MI by elevating the expression of high mobility group box 1, a nuclear protein from necrotic cells that can induce an inflammatory response [52]. Lastly, *P. gingivalis*-associated production of IL-17A might play an essential role in the pathology of MI, as IL-17A was found to exacerbate ventricular remodeling after acute MI in mice [50].

### 2.3. Abdominal Aortic Aneurysms (AAAs)

AAA is a permanent localized widening of the abdominal aorta. A prospective study in 2004 first detected periodontal bacteria by PCR in aneurysmal specimens from AAA patients, and the detection rate of *P. gingivalis* was 85%, the highest among all detected periodontal pathogens [53]. Subsequently, further experiments in mice and rats indicated that both intravenous and subcutaneous injection of *P. gingivalis* resulted in a significant increase in the abdominal aortic diameter or intraluminal thrombus (ILT) expansion [54,55].

*P. gingivalis* mainly promotes AAA progression via three key processes: inflammation, proteolysis, and SMC apoptosis. Firstly, recognition of *P. gingivalis* by TLR-2 induces an inflammatory response and accelerates the development of experimental AAAs [55]. *P. gingivalis* may also promote neutrophil recruitment and activation in AAA thrombus, along with the formation of neutrophil extracellular traps (NETs) in ILT, ultimately stimulating AAA growth [56]. Secondly, MMP expression in infiltrating inflammatory cells is increased when stimulated by *P. gingivalis*, which suggests more active proteolytic activities and neutrophil chemotaxis in the luminal layer of ILT [54]. Lastly, *P. gingivalis* infection resulted in a significant elevation of myeloperoxidase–DNA complexes in aneurysm samples and plasma [56], thereby enhancing oxidative stress and ultimately contributing to AAA pathogenesis through MMP dysregulation and SMC apoptosis [57].

### 2.4. Hypertension

Hypertension, currently defined as high systolic blood pressure (BP) (≥140 mmHg) and/or high diastolic BP (≥90 mmHg), is a growing public health problem worldwide. In recent years, a positive link between periodontitis and hypertension has been reported by several epidemiological studies. The results show that patients with diagnosed periodontitis (both moderate-severe and severe) have an increased likelihood of developing hypertension [58]. Moreover, clinical trials with periodontal intervention showed a significant decrease in systolic BP and diastolic BP after periodontal therapy [59]. This evidence preliminarily confirms the role of periodontitis in hypertension. Furthermore, in vivo studies have shown that intraperitoneal injection of *P. gingivalis* exacerbated the hypertensive response to angiotensin II in mice [60], suggesting an adverse effect of *P. gingivalis* on hypertension.

Hypertension is mainly caused by functional changes in blood vessels, such as increased vasoconstrictor responses and endothelial dysfunction. Its etiology is complicated, with no simple mechanism that entirely explains it, and inflammation and the immune response are both implicated. *P. gingivalis* has been reported to affect these processes and thereby promote hypertension. It was shown that repeated exposure of live *P. gingivalis* or bacteria LPS induced the release of pro-inflammatory CKs and angiotensin II in human coronary artery endothelial cells, together with *P. gingivalis*-associated mediators of systemic inflammation (such as CRP, IL-6, TNF-α), contributing to both endothelial dysfunction and the development of arterial hypertension [61]. Recently, an animal study supported the hypothesis that the Th1 immune response induced by *P. gingivalis* antigens is response for elevated BP [60]. Moreover, *P. gingivalis* can induce high expression of endothelial cell adhesion molecules, platelet aggregation, and SMC proliferation, thus impairing vasomotor function [33,34,42].

## 3. Oncology

As the major cause of death across the world, cancer is characterized by uncontrolled cell growth due to aberrant cell cycle progression. Periodontal disease patients were found to have a greater risk of cancer, with a small but significant correlation [62]. With further research, positive connections between *P. gingivalis* and digestive system cancer have been reported. Intriguingly, increased mortality from *P. gingivalis*-related digestive system cancer was also exhibited in otherwise healthy individuals, providing evidence for a direct role that is independent of periodontal disease. Additionally, the authors identified *P. gingivalis* as a valuable microbial marker of mortality risk in digestive system cancer [63].

### 3.1. Oral Cancer

Oral squamous cell carcinoma (OSCC) often occurs in the tongue, floor of the mouth, buccal mucosa, and gingiva. A meta-analysis revealed that periodontitis increased the risk of OSCC by nearly 2-fold [64]. Furthermore, *P. gingivalis* was shown to increase the chance of OSCC, and its colonization in tumor tissues has reduced patient survival [65]. The detection of *P. gingivalis* by immunohistochemical staining was more than 33% higher in gingival carcinoma tissues than in normal gingival tissues [66]. Afterwards, in vivo experiments further confirmed the negative effect of *P. gingivalis* in OSCC. It was found that *P. gingivalis* accelerated OSCC progression in an immune microenvironment through the secretion of C-C motif chemokine 2, chemokine (C-X-C motif) ligand 2, IL-6, and IL-8 from infected oral dysplastic keratinocytes to recruit myeloid-derived suppressor cells [65]. In addition, oral administration of *P. gingivalis* promoted 4-nitroquinoline-1-oxide-induced tongue tumorigenesis and aggravated the disturbance of fatty acid metabolism during oral carcinoma progression [3].

**Figure 2:**
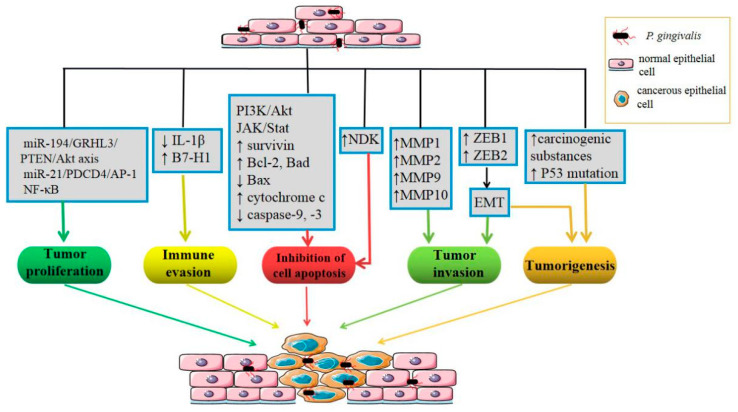
Cancer-associated processes in which *Porphyromonas gingivalis* may be implicated.

Large studies have explored the mechanisms of *P. gingivalis* in OSCC, including epithelial–mesenchymal transition (EMT) of oral epithelial cells, the inhibition of epithelial cell apoptosis, the promotion of immune evasion, the proliferation and invasion of tumor cells, and so on (Figure 2). First, *P. gingivalis* upregulates the levels of zinc-finger E-box-binding homeobox proteins (ZEB1 and ZEB2), which are transcription factors that regulate EMT through GSK-3β [67] and β-catenin/forkhead box-O1 (FOXO1), respectively [68]. A review by Olsen provided a summary of evidence related to *P. gingivalis*-induced EMT of OSCC cells and human primary oral epithelial cells [69]. Second, *P. gingivalis* modulates epithelial cell apoptosis via multiple anti-apoptotic/survival pathways, including the activation of the PI3K/Akt and JAK/Stat pathways, release of survivin, upregulation of anti-apoptotic Bad and Bcl-2, downregulation of pro-apoptotic Bax, and inhibition of cytochrome c release and caspase-9 and caspase-3 activation [70,71]. *P. gingivalis* infection upregulates miR-203, which directly inhibits suppressor of cytokine signaling 3 and leads to increased Stat3 activation [72], which may also be involved in the anti-apoptotic mechanism of the epithelium, given the role of Jak/Stat mentioned above. In addition, nucleoside diphosphate kinase (NDK), an ecto-ATPase secreted by intracellular *P. gingivalis*, confers epithelial cells with an anti-apoptotic phenotype by binding to and phosphorylating HSP27, which inhibits cytochrome c release and caspase-9 activation [73]. NDK from *P. gingivalis* can also scavenge ATP and inhibit P2X7-mediated host-cell apoptosis [74]. Moreover, *P. gingivalis* infection promotes a prosurvival phenotype in human primary oral epithelial cells by regulating cyclins and p53 [75]. *P. gingivalis*-induced reactive oxygen species (ROS) activates the multipurpose transcriptional regulator FOXO1 via JNK signaling and then initiates an anti-apoptotic program in epithelial cells [76]. Third, internalized *P. gingivalis* upregulates the expression of B7-H1 and B7-DC receptors on oral cancer cells via a receptor-interacting serine/threonine-protein kinase 2-dependent mechanism, contributing to the escape of tumor cells from immunosurveillance [77]. Furthermore, NDK from *P. gingivalis* has antagonist effects on ATP activation of P2X7 receptors, leading to reduced IL-1β production in epithelial cells, thereby promoting the immune evasion of tumor cells [78]. Fourth, *P. gingivalis* promotes the proliferation of OSCC cells by regulating the expression of cyclin D1 through the miR-21/PDCD4/AP-1 negative signaling pathway [79]. Fifth, *P. gingivalis*-infected OSCC cells can increase invasiveness through EMT-like changes [80]. In addition to EMT, *P. gingivalis* may have the ability to induce proMMP9 expression via ERK1/2-Ets1, p38/HSP27, and PAR2/NF-kB pathways, which subsequently activate MMP9, promoting OSCC cell invasion [81]. *P. gingivalis* exposure also increases the invasive ability of oral cancer cells via the upregulation of MMPs in an IL-8-dependent fashion, including MMP-1, MMP-2, and MMP-10 [80,82]. Beyond the mechanisms mentioned above, it has been reported that inflammatory mediators elicited by *P. gingivalis* could induce cell proliferation, mutagenesis, oncogene activation, angiogenesis, and immunosuppression, thus facilitating the development of OSCC [83].

### 3.2. Esophageal Cancer

Esophageal cancer is one of the most aggressive cancers in the world. A positive correlation was found between *P. gingivalis* and higher esophageal squamous cell carcinoma (ESCC) risk. The expression of whole *P. gingivalis* antigen and unique Kgp, along with 16S rDNA, was much higher in cancerous tissues compared with adjacent and normal tissues from ESCC patients [84]. Moreover, *P. gingivalis* was inversely related to the ESCC survival rate [84]. Patients with higher levels of *P. gingivalis* IgA or IgG tended to have a worse prognosis [85], indicating the adverse role of *P. gingivalis* in ESCC.

In order to investigate the underlying mechanisms, a series of in vitro experiments has been carried out. *P. gingivalis* dehydrogenates ethanol to its carcinogenic derivative, acetaldehyde, at levels that are capable of inducing DNA damage, mutagenesis, and excessive proliferation of the epithelium. Carcinogen metabolism by *P. gingivalis* may therefore be a possible mechanism [84]. Further, Yuan et al. highlighted the ability of *P. gingivalis* to evade immune surveillance, and they determined that the blockade of immune-checkpoint B7-H4 and lysine demethylase 5b in ESCC conferred resistance against *P. gingivalis* infection and tumor challenge, proposing them as potential therapeutic targets for controlling *P. gingivalis* infection and its associated neoplasia [86]. Furthermore, *P. gingivalis* can enhance ESCC cell proliferation and metastasis by triggering the NF-κB signaling pathway [87] or via the miR-194/GRHL3/PTEN/Akt axis [88].

### 3.3. Pancreatic Cancer

Pancreatic cancer, one of the most rapidly fatal diseases, represents one of the primary causes of cancer-related mortality. People with a history of periodontal disease showed a higher risk of developing pancreatic cancer, which was up to 1.64 times higher relative to those without periodontal disease [89]. Subsequently, researchers aimed to explore the potential relationship between oral bacteria and pancreatic cancer. For example, in a large European prospective cohort study, investigators examined 25 oral bacteria and found that higher levels of serum *P. gingivalis* antibodies were associated with a two-fold increased risk of pancreatic cancer [90]. In another large-scale and long-term study, researchers sequenced DNA extracted from the saliva samples of participants and found that individuals who were positive for *P. gingivalis* had a 59% greater risk of developing pancreatic cancer compared with those in whom the species was undetected [91]. This evidence suggests that there may be a relationship between *P. gingivalis* and pancreatic cancer.

Some possible mechanisms have been proposed. *P. gingivalis* infection could induce differential expression of TLR pathway-associated genes [92], and TLR activation has critical protumorigenic effects on pancreatic carcinoma [93]. Additionally, *P. gingivalis* invasion leads to p53 activation, a tumor suppressor gene, whose mutation rate is higher in pancreatic cancer and whose abnormal expression plays an important role in pancreatic tumorigenesis [94]. Thus, mutations in p53 may serve as a bridge that connects *P. gingivalis* and pancreatic cancer development. Although partly clarified, further observations are needed to elucidate how *P. gingivalis* participates in the progression of pancreatic cancer.

## 4. Neurology

Mental and neurological disorders, involving alterations in brain or nervous system functions, together with perception and response to the environment, have afflicted the global human population. The relationship between periodontitis and neuropsychiatric disorders has recently captured the interest of scientists. Epidemiological evidence from several studies has indicated that chronic periodontitis and *P. gingivalis* infection significantly elevate the risk of neuropsychiatric disorders, such as Alzheimer’s disease [95] and depression [96].

### 4.1. Alzheimer’s Disease (AD)

AD is a neurodegenerative disease. It is the most common reason for dementia and is becoming a major health problem in aging societies worldwide. A historical cohort study found that chronic periodontitis patients had an elevated risk of AD [95], and *P. gingivalis* DNA, LPS [97], and gingipains [4] have been identified in AD brains. Parallel epidemiological studies and animal experiments also strengthen support for the possible relevance of *P. gingivalis* in AD pathogenesis. *P. gingivalis* exposure induced AD-like phenotypes in mice, which presented as microglia-mediated neuroinflammation, β-amyloid (Aβ) accumulation in neurons, impaired cognitive function, and a reduction in learning and memory [98,99].

**Figure 3:**
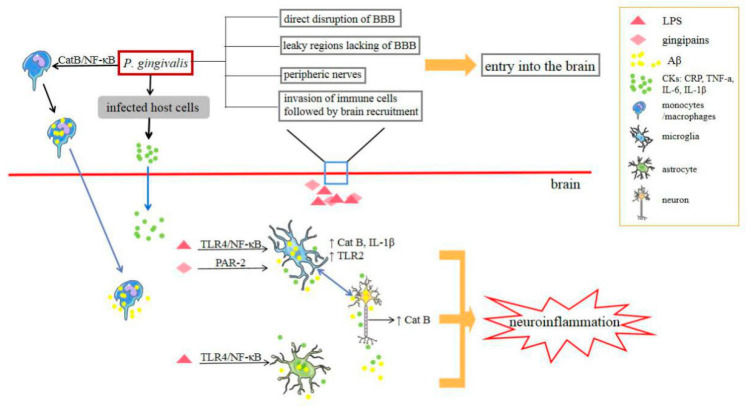
Scheme for the presumed mechanisms by which *Porphyromonas gingivalis* may access the brain and its pathogenic mechanisms. BBB: blood–brain barrier.

The pathology of AD has three major hallmarks: Aβ plaques, neurofibrillary tangles, and microglia-mediated neuroinflammation. *P. gingivalis* may be involved in the progression of AD through these processes (Figure 3). Cathepsin B (CatB) is crucial for Aβ deposition and the mediation of neuroinflammation. Intraperitoneal injection of *P. gingivalis* LPS into middle-aged WT mice significantly increased CatB expression in both microglia and neurons, and *P. gingivalis* LPS-induced AD-like phenotypes were found to occur only in a CatB-dependent manner [98]. Furthermore, in *P. gingivalis*-infected mice, Aβ accumulated in inflammatory monocytes/macrophages, mainly based on CatB/NF-κB signaling activation, and this is the first evidence to suggest that inflammatory monocytes/macrophages act as the source of peripheral Aβ when exposed to *P. gingivalis* [100]. Therefore, considering the pivotal role of CatB, it has been regarded as a potential therapeutic target for preventing *P. gingivalis*-associated cognitive decline in AD [98,100]. In addition, hippocampal neurons had significantly increased mean mRNA levels of amyloid precursor protein and CatB when treated with conditioned medium from *P. gingivalis* LPS-treated WT primary microglia, but not when treated with *P. gingivalis* LPS directly [98]. Accordingly, the pivotal, detrimental role of *P. gingivalis* against microglia is involved in the pathogenesis and development of AD. A recent review indicated that *P. gingivalis* may affect microglia and genes such as apolipoprotein, clusterin, CD33, and complement receptors to cooperatively promote the neurodegeneration that is typical of AD [101]. Furthermore, *P. gingivalis* LPS might induce neuroinflammation and cognitive impairment in mice via the TLR4/NF-κB signaling pathway [99]. In addition to the direct role of *P. gingivalis*, the release of inflammatory molecules may also be involved. Various host cells infected with *P. gingivalis* reportedly release a set of CKs, such as TNF-α, IL-1, IL-6, and IL-8, in their immune response [102]. Pro-inflammatory mediators could reach the central nervous system via hematoencephalic barrier-free areas and fenestrated capillaries or by regulating blood–brain barrier permeability [103]. Systemic inflammation, including inflammatory mediators such as CRP, TNF-a, IL-6, and IL-1β, may also increase the risk and progression of cognitive decline and AD. In contrast to the above-described studies, Liu et al. focused on gingipains, Rgp and Kgp. They revealed that gingipains cooperatively contributed to the cell migration of microglia towards the infected site and induced neuroinflammation through the proteolytic activation of protease-activated receptor 2, and the subsequent activation of PI3K/Akt and MEK/ERK pathways may also play fundamental roles in this process [104]. In addition, researchers have proposed that *P. gingivalis* may also be involved in the progress of AD by contributing to the suppression of the host’s adaptive immune system. *P. gingivalis* could prevent the entry of immune cells into the brain, increase blood–brain barrier permeability, and inhibit the local IFN-γ response. The scarcity of adaptive immune cells in AD neuropathology further implies that *P. gingivalis* residing in the brain may impair clearance of Aβ and induce immunosuppression [5].

### 4.2. Depression

Depression, a psychological disorder, is affecting increasingly more people around the world. It is easily affected by social psychology. Previous studies have mainly focused on the psychosocial effects (such as embarrassment, isolation, loneliness) of tooth loss, poor oral hygiene, and halitosis [105]. It has been reported that individuals with periodontitis have a higher incidence of subsequent depression [96]. Scholars have attempted to explore the biological mechanisms through which periodontitis contributes to depression. Wang et al. successfully produced depression-like phenotypes in mice after *P. gingivalis* oral colonization or *P. gingivalis* LPS intraperitoneal injection. The proposed underlying mechanism was that *P. gingivalis* induced astrocyte activation and downregulated neurotrophic factor receptor p75 expression in a TLR4-dependent fashion, ultimately inhibiting the maturation of the brain derived of neurotrophic factor. This study has provided the first experimental evidence that *P. gingivalis* could be a risk factor for depression [106]. More studies are needed.

## 5. Respirology

Respiratory diseases are among the most common pathologies in humans across the world. Many published epidemiological studies have reported a positive link between periodontitis and respiratory diseases [107] through the possible aspiration of oral bacteria from the oropharynx into the lower respiratory tract as a result of a swallowing disorder. Among the different periodontitis-related respiratory diseases, we mainly discuss pneumonia and chronic obstructive pulmonary disease here.

### 5.1. Pneumonia

Lower respiratory tract infections, including pneumonia, were the fourth highest cause of death worldwide in 2016 [108]. A recently published meta-analysis validated a positive correlation between periodontitis and pneumonia [107], and dental treatment has been regarded as a safe and effective means of preventing lower respiratory tract infection in intensive care patients [109]. Moreover, mice that were intratracheally infected with *P. gingivalis* developed severe bronchopneumonia and lung abscess [23,110].

Studies have investigated the correlation between *P. gingivalis* and pneumonia, and some key findings have been reported. Evidence from in vivo experiments suggests that gingipains are essential factors in mediating aspiration pneumonia due to *P. gingivalis* infection, and the authors recommended it as a useful adjunct treatment site [23]. Interestingly, TLR-2 has been demonstrated to confer protection against acute pulmonary infection with *P. gingivalis* [110]. Furthermore, *P. gingivalis* might affect pneumonia progression through interactions with other respiratory pathogens. Co-infection of *P. gingivalis* and H1N1 in lung epithelial cells promoted the production of inflammatory CKs and NO, resulting in increased levels of apoptosis via the Bcl-2/Bax/caspase-3 pathway [111]. *P. gingivalis* can modulate *Pseudomonas aeruginosa*-induced respiratory epithelial cell apoptosis via the STAT3 signaling pathway by causing transient inhibition and, ultimately, exerting promoting effects [112].

### 5.2. Chronic Obstructive Pulmonary Disease (COPD)

COPDs are a group of diseases characterized by airflow limitation in the airway and include chronic bronchitis and emphysema. A positive relationship between periodontitis and COPD was reported [107], and periodontal therapy was found to improve lung function and reduce the frequency of COPD exacerbation in COPD patients [113]. Through 16S rDNA-based metagenomic analysis, *P. gingivalis* was found in tracheal aspirates from patients with severe acute exacerbation of COPD [114], suggesting a possible role for the bacteria in this disease. Studies have indicated that *P. gingivalis*-associated enzymes in saliva (such as proteases) may alter mucosal surface adhesion receptors, destroy salivary pellicles on pathogenic bacteria, or degrade the salivary pellicles on mucosal layer, all of which promote the colonization of respiratory pathogens in the respiratory epithelium [115]. These findings suggest that *P. gingivalis* plays a role in COPD progression by modulating the colonization of respiratory pathogens.

## 6. Metabolism

Metabolic diseases refer to a class of disorders caused by the abnormal metabolism of glucose, proteins, or lipids. Their prevalence has markedly increased across the world in the last few decades. Many studies have linked *P. gingivalis* to several metabolic diseases, especially diabetes and nonalcoholic fatty liver disease.

### 6.1. Diabetes

Diabetes, a series of chronic metabolic disorders characterized by hyperglycemia, is the result of defects in insulin secretion, insulin action, or both. Considerable epidemiologic evidence has shown a two-way interrelationship between periodontitis and diabetes, and many researchers regard periodontal infection as a risk factor for diabetes progression. Diabetic individuals with periodontitis were found to have a significantly higher prevalence of diabetes-related complications [116]. Furthermore, patients with diabetes had a reduction of 0.4% in glycosylated hemoglobin after non-surgical periodontal therapy [117]. Further animal experiments have confirmed that *P. gingivalis* or its components can induce insulin resistance in mice [118,119,120,121], which is one of the vital pathogeneses of type 2 diabetes.

Among the potential *P. gingivalis*-related mechanisms involved in the course of diabetes, insulin resistance is considered to be the most important. *P. gingivalis* infection increased systemic inflammation, especially in adipose tissue, through the induction of endotoxemia [119], alteration of gut microbiota [122], or an impaired regional adaptive immune response [118], ultimately resulting in insulin resistance. Moreover, inflammation and elevated levels of inflammatory markers, such as CRP and IL-6, can induce insulin resistance [123]. Thus, increased levels of systemic pro-inflammatory CKs initiated by *P. gingivalis* may promote insulin resistance. *P. gingivalis* LPS induced pro-inflammatory adipokine secretion and oxidative stress in adipocytes through the regulation of TLR-mediated pathways and redox enzymes and contributed to obesity-associated insulin resistance [124]. A recent study in 2020 indicated that *P. gingivalis* aggravated high-fat diet (HFD)-induced insulin resistance in mice via its biosynthesis of branched-chain amino acids, which can activate the mammalian target of rapamycin and downstream genes, which leads to the dephosphorylation of insulin receptor substrate 1 [121]. Furthermore, *P. gingivalis* OMVs were loaded with gingipains and translocated to the liver in mice, leading to the attenuation of insulin sensitivity and inhibition of hepatic glycogen synthesis, partly by attenuating the Akt and GSK-3β pathway [120]. Importantly, potential mechanisms other than insulin resistance should be examined. Higher colonization levels of *P. gingivalis* are reported to be linked to higher prediabetes prevalence among diabetes-free adults [125]. *P. gingivalis* LPS stimulated insulin secretion by pancreatic β-cells and had significant implications in the progression of β-cell compensation in prediabetes in subjects with periodontitis [126]. Moreover, oral administration of *P. gingivalis* induced the translocation of *P. gingivalis*/gingipains to the pancreas in mice, leading to significant changes in islet architecture and β-cell apoptosis, which may be involved in the development of prediabetes [127]. Lastly, *P. gingivalis* attenuated the phosphorylation and translocation of FOXO1, which is regulated by insulin in HepG2 cells, thereby increasing hepatic gluconeogenesis [128] and potentially leading to elevated blood glucose.

### 6.2. Nonalcoholic Fatty Liver Disease (NAFLD)

NAFLD is a hepatic manifestation of metabolic syndrome characterized by abnormal accumulation of fat. Epidemiological evidence in the last decade has supported a positive correlation between periodontitis and high prevalence odds of NAFLD. In NAFLD patients, liver function parameters (ALT and AST) were improved after they underwent periodontal therapy [129]. A potential link between *P. gingivalis* and NAFLD has also been shown. The detection frequency of *P. gingivalis* DNA (46.7%) was markedly higher in NAFLD patients compared with control subjects [129]. *P. gingivalis* was also immunohistochemically detected (52.5%) in the liver of nonalcoholic steatohepatitis (NASH) patients, and NASH patients with *P. gingivalis* infection had higher fibrosis scores than those who did not [130]. Similarly, antibodies against *P. gingivalis* with type IV fimbriae tended to increase with fibrosis progression among NASH patients [131]. Moreover, in vivo studies have indicated that *P. gingivalis* infection can exacerbate HFD-induced NAFLD/NASH progression [119,129,130,131,132].

Over the past decade, potential underlying mechanisms that connect *P. gingivalis* to NAFLD have been explored. The involvement of *P. gingivalis* in the pathogenesis of NAFLD in mice may occur through the activation of the *P. gingivalis* LPS-associated TLR2 pathway and inflammasomes [130], the severe disruption of fatty acid metabolism, and an increase in the monounsaturated/saturated fatty acid ratio [131]. Moreover, insulin resistance is currently considered to be one of the important pathogenesis mechanisms of NAFLD. Endotoxemia caused by *P. gingivalis* injection aggravated NAFLD in mice, along with impaired glucose tolerance and insulin resistance [119]. We previously described mechanisms of *P. gingivalis* related to insulin resistance that may also have effects on NAFLD. This was also mentioned in related review [6]. A recent study in 2020 further explored the mechanisms of liver fibrosis. The authors investigated the interaction between *P. gingivalis* and hepatic stellate cells (HSCs), which act as effector cells of hepatic fibrosis. The presented data suggested that *P. gingivalis* odontogenic infection could play a role in aggravated fibrosis in a NASH mouse model through HSCs activation caused by the production of TGF-β1 and galectin-3 in HSCs and hepatocytes [132]. Other putative explanations that have been proposed are the alteration of the gut microbial composition due to swallowed *P. gingivalis*, along with the production of CKs and ROS triggered by *P. gingivalis* [6]. Despite recent progress, there is comparatively little research on this subject. Further studies are still warranted to explore its biological mechanisms. On the other hand, because all of these studies are cross-sectional and include heterogeneity, further large-scale prospective cohort studies are highly recommended to verify the association.

## 7. Obstetrics

Adverse pregnancy outcomes (APOs) refer to any maternal, fetal, and/or neonatal complications that occur during pregnancy, labor, and/or the postpartum period. Epidemiologic evidence has consistently suggested a positive correlation between periodontitis and APOs [133]. Furthermore, *P. gingivalis* DNA and antigens have been found in the placenta, umbilical cord [134], and amniotic fluid [135]. A positive association has been found between the presence of *P. gingivalis* within the placenta or umbilical cord of pregnant women and many pregnancy complications, including preterm birth (PB), preeclampsia, and pregnancy-related hypertensive disorders [134,135,136]. These results suggest the involvement of *P. gingivalis* in APOs through direct invasion and damage to utero-placental tissues. Subsequently, various rodent models confirmed that *P. gingivalis* infection can induce diverse utero-placental pathologies (such as endometrial arteritis, mild chorioamnionitis, and utero-placental hemorrhage with structural disorder of placenta) and cause a diverse array of APOs, including fetal growth restriction (FGR), low birth weight (LBW), and PB [137,138,139].

The direct dissemination of *P. gingivalis* to the placenta and its pathogenicity mechanisms have been proposed in many animal experiments. It has been reported that *P. gingivalis* could translocate to local placental tissues in rats and enhance the expression of Fas, FasL, and TLR2, leading to PB and LBW [137]. *P. gingivalis* infection in pregnant mice induced a fetus-specific placental immune response and increased the placental Th1/Th2 cytokine ratio (increased expression of IFN-γ, IL-2, and IL-12, decreased expression of IL-4 and IL-10), which may be related to the occurrence of FGR [138]. The same research team also found that *P. gingivalis* induced a maternal immune response and enhanced FGR in mice, with an elevation in the maternal serum TNF-α and a decrease in IL-10 [139]. Furthermore, *P. gingivalis* LPS directly or indirectly upregulated inflammatory factors such as TNF-α, IL-8, and cyclooxygenase 2 via the production of galectin-3 in the placenta, inducing PB [140]. Researchers have also explored the impacts of *P. gingivalis* on trophoblast cells. *P. gingivalis* can invade human extravillous trophoblasts and induce G1 arrest and cell apoptosis, which may involve ERK1/2 and DNA damage response pathways, thereby disrupting the maintenance of pregnancy [141]. The activation of complex signaling networks, for example, the HSP27/p21 pathway via p53/p38 and JNK, may also contribute to G1 arrest and cell apoptosis [94]. Other mechanisms have been proposed and summarized in detail previously, including *P. gingivalis*’ persistence and survival within utero-placental tissues via sophisticated invasive strategies and immune response evasion, development of polymicrobial dysbiosis and synergy with other commensal bacteria to increase the overall microbial burden at the maternal–fetal interface, increased expression of acute-phase proteins such as CRP and pentraxin3, excessive generation of ROS, increased production of fetal adrenal cortisone, and fetal stress [7].

## 8. Rheumatology

As a chronic autoimmune disease, rheumatoid arthritis (RA) is characterized by persistent inflammatory synovitis and progressive degeneration of cartilage and bone. A meta-analysis found a higher RA prevalence in periodontitis patients compared with controls [142], and non-surgical periodontal therapy in individuals with RA and periodontitis was associated with improvements in markers of disease activity in RA [143]. Many periodontal bacteria, particularly *P. gingivalis*, were detected in synovial fluid from patients with RA at the DNA level (42.1%) [144]. Subsequently, a great deal of preclinical experiments in different animal models revealed that *P. gingivalis* increased the incidence and progression of RA [145,146,147,148].

The etiology of RA is very complicated. Autoantibodies against citrullinated proteins are confirmed to be one of its pathological bases. Protein citrullination is carried out by peptidyl-arginine deiminases (PAD), and, so far, *P. gingivalis* is the only microorganism known to express the PAD enzyme. A previous review investigated and proposed that *P. gingivalis* PAD may set in motion a chain of events that break immunotolerance to citrullinated proteins, leading to the development of RA [8]. Treatment with a PAD-deficient strain effectively reduced the extent of experimental arthritis in animals compared with those infected with wild-type *P. gingivalis* [147]. Additionally, oral inoculation of *P. gingivalis* prior to RA activated the immune system by inducing a TLR2- and IL-1-driven Th17 cell response, which suggests that exposure to *P. gingivalis* may accelerate RA progression predominantly through Th17-related pathways [145]. Furthermore, it has been proposed that the promotion of C5a generation, the induction of NETosis, osteoclastogenesis [149], and orally administered *P. gingivalis*, which alters the gut immune system and gut microbiota composition [146], may also be implicated in the progression of RA. Inflammatory arthritis alters the intestinal inflammatory response with the downregulation of some protective mediators, including resolvin D5_n-3 DPA_, which weakens the intestinal barrier function and facilitates a switch in *P. gingivalis* behavior to the pathogenic tendency, thus further promoting gut bacterial translocation and increasing joint inflammation [150]. 

## 9. Strategies for Controlling *P. gingivalis* for the Potential Treatment of Systemic Diseases

Given the inseparable link between *P. gingivalis* and several systemic diseases, many researchers naturally want to explore whether controlling *P. gingivalis* can be regarded as a feasible disease management strategy. Proposed approaches include broad-spectrum antibiotics and specific inhibitors that target *P. gingivalis*. Firstly, administration of doxycycline or metronidazole resulted in a significant reduction in the percentage of atherosclerotic lesions in *P. gingivalis*-inoculated mice [151,152]. Additionally, Dominy and colleagues made a new breakthrough, as they designed an orally administered small molecule that targets gingipains [4]. Gingipain inhibitors have been found to block gingipain-induced neurodegeneration, rescue neurons in the hippocampus, and significantly reduce the *P. gingivalis* load and Aβ production in the mouse brain, suggesting that it could be valuable for treating *P. gingivalis* brain colonization and neurodegeneration in AD [4]. More recently, pre-immunization with purified recombinant Rgp domains alleviated experimental arthritis in rats [153]. Pretreatment with inhibitors to block the proteolytic activity of gingipains in mice significantly relieved the symptoms and reduced the mortality of pneumonia, indicating that inhibition of gingipains may be a useful adjunct in the treatment of *P. gingivalis*-mediated aspiration pneumonia [23]. Besides gingipains, sublingual immunization with a recombinant vaccine that targets *P. gingivalis* HSP60 or nasal immunization with OMVs prior to *P. gingivalis* injection also significantly reduced AS lesion formation in ApoE-deficient mice, and it may be an effective strategy for preventing naturally occurring or *P. gingivalis*-accelerated AS [154,155]. Targeting the main virulence factors of *P. gingivalis* (such as gingipains) by pre-immunization or, if possible, small-molecule inhibitors may reduce the ability of the bacteria to translocate to remote tissues and instigate and/or exacerbate pathology in some chronic inflammatory diseases [153]. Despite the positive results mentioned above, more research is still warranted, which may provide novel therapeutic strategies for some diseases.

## 10. Conclusions

**Figure 4:**
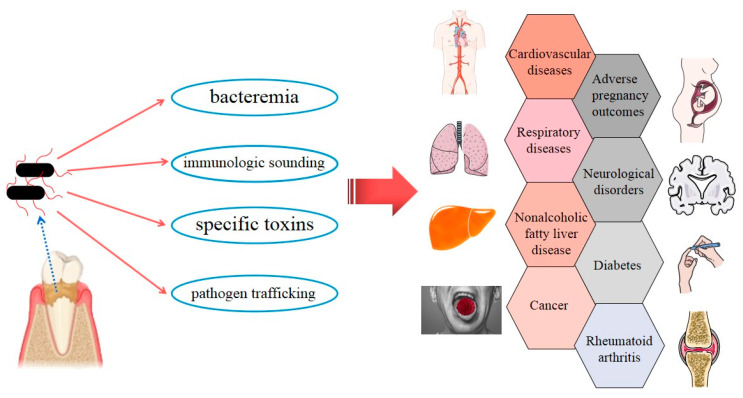
Strategies by which *Porphyromonas gingivalis* can invade the whole body, along with simple a schematic representation of *Porphyromonas gingivalis*-associated systemic diseases.

The latest studies have tended to focus on exploring possible direct and indirect links between *P. gingivalis* and certain systemic diseases, especially AD and AS, and an increasing amount of evidence has been accumulating. *P. gingivalis* invades the human body and influences general health in four main ways (Figure 4). (i) Bacteremia: The direct invasion of *P. gingivalis* in epithelium, endothelial cells, and subepithelial tissues has been demonstrated [156]. Transient bacteremia of *P. gingivalis* frequently occurs during daily activities, such as toothbrushing, chewing, and flossing, and after dental treatment procedures [20], which enable the bacteria to enter the rest of the body and take part in local pathogenesis. (ii) Immunologic sounding: Persistent local infection caused by *P. gingivalis* induces the upregulation of inflammatory cascades involving IL-1, IL-6, IL-8, TNF-α, CRP, and IFN [102]. A low-level, long-term systemic inflammatory status might be implicated in the pathology of systemic disorders. (iii) Specific toxins of *P. gingivalis*, such as gingipains and OMVs, have been detected at multiple body sites (such as the brain and liver) and play pivotal roles in the progression of local diseases [4,120]. (iv) Pathogen trafficking: Direct infection and internalization in host immune cells, such monocytes, and subsequent recruitment to tissues throughout the whole body may be another pathogenic strategy of *P. gingivalis* [4].

In this article, we comprehensively summarize the adverse effects of *P. gingivalis* on multiple systems and a variety of diseases, from extensively studied fields (CVDs, cancer, APOs, etc.) to emerging areas (AD, NAFLD, depression, etc.). Some of the clearer and more direct mechanisms are discussed here. However, many of the exact mechanisms are not fully clear yet, and a few conclusions are controversial. Current evidence emphasizes that promoting oral health should be encouraged as an indispensable part of a healthy lifestyle to reduce the burden of global chronic noncommunicable and communicable diseases [157], which was an initiative of the World Health Assembly as early as 2007. There is currently no doubt that periodontitis is certainly preventable and controllable, as is *P. gingivalis*, and should be regarded as a modifiable factor for diseases. Reducing the load of *P. gingivalis* through periodontal intervention has potential benefits for oral health and a direct or indirect positive impact on overall health, even if it prevents the possibility of such a connection. We should be aware of the risk and address it as soon as possible to avoid threatening our health. Moreover, interrelations between *P. gingivalis* and other oral pathogens (such as *Actinobacillus actinomycetemcomitans* and *Fusobacterium nucleatum*), together with gut microbiota, are another subject of intensive study.
